# Elevated COMMD1 Contributes to Cardiomyocyte Copper Efflux in Chronic Myocardial Ischemia: Insights From Rhesus Monkey

**DOI:** 10.1111/cpr.70016

**Published:** 2025-03-03

**Authors:** Chen Li, Da Li, Xia Cheng, Xiaoli Yuan, Ning Du, Xin Liao, Xiaorong Feng, Jie Yao, Chenglong Li, Chengxia Xie, Mu Yang

**Affiliations:** ^1^ Department of Experimental Research, Sichuan Clinical Research Center for Cancer, Sichuan Cancer Hospital & Institute, Sichuan Cancer Center University of Electronic Science and Technology of China Chengdu Sichuan China; ^2^ Department of General Internal Medicine, Sichuan Clinical Research Center for Cancer, Sichuan Cancer Hospital & Institute, Sichuan Cancer Center University of Electronic Science and Technology of China Chengdu China; ^3^ Chengdu Customs Technology Center Chengdu China; ^4^ Department of Pharmacy, Deyang People's Hospital Affiliated Hospital of Chengdu University of Traditional Chinese Medicine Deyang China; ^5^ Department of Laboratory Medicine, West China Hospital Sichuan University Chengdu China

**Keywords:** chronic myocardial ischemia, COMMD1, copper efflux, XIAP

## Abstract

Copper deficiency, commonly observed in myocardial infarction, leads to cardiomyocyte loss and cardiac dysfunction, yet the mechanism driving copper efflux remains unclear. To further elucidate the relationship between copper transporters and cardiac copper efflux during chronic myocardial ischemia, a rhesus monkey model was established by performing the permanent ligation of the left anterior descending coronary artery. A dramatic decrease in copper concentration within ischemic cardiomyocytes was observed alongside declining cardiac function. Among major copper transporters, COMMD1 and ATP7B were significantly upregulated in the ischemic myocardium. COMMD1 was specifically localised in cardiomyocytes undergoing copper efflux, whereas increased ATP7B was restricted to cardiac fibroblasts. This indicates that elevated COMMD1 regulates copper efflux in cardiomyocytes during chronic myocardial ischemia, functioning independently of its interactions with P‐type ATPase transporters. Given the discrepancy between RNA and protein levels of COMMD1 in ischemic myocardium, post‐translational modification is likely responsible for regulating COMMD1 expression. We found that the copper‐binding protein with E3 ubiquitin ligase activity, XIAP, augmented before the rise in COMMD1 expression within ischemic cardiomyocytes. Excessive XIAP specifically interacted with COMMD1 to enhance its protein levels under copper‐deprivation conditions and vice versa. Overall, our findings reveal a positive feedback loop among XIAP, COMMD1 and copper, highlighting the intricate interplay between XIAP and COMMD1 in regulating copper efflux in cardiomyocytes. This loop sets the stage for further investigation into therapeutic strategies to manage copper homeostasis in chronic myocardial ischemia.

## Graphical abstract

Elevated COMMD1 contributes to copper efflux in cardiomyocytes during chronic ischaemia in rhesus monkey hearts, regulated by XIAP through post‐translational modification. Our findings suggest a positive feedback loop among XIAP, COMMD1 and copper efflux, contributing to ischaemic cardiomyocyte loss.

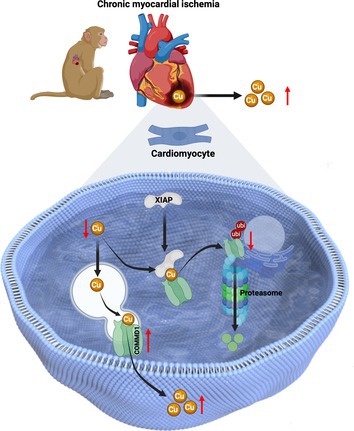

## 1 Introduction

Coronary artery occlusion is the leading cause of ischemic heart disease, resulting in approximately 18 million cardiovascular deaths worldwide annually [1]. Due to the reduction of blood supply, hypoxia and nutrient deprivation could induce massive cardiomyocyte death within the ischemic area, thereby impairing the contractile function of the heart [2]. As a consequence of cardiomyocyte loss, innate immunity leads to the transformation of fibroblasts and collagen deposition, which occur in the maintenance of structural integrity but further deteriorate myocardial contractile function [3, 4, 5, 6]. Although reperfusion by percutaneous coronary intervention and pharmacological treatments has been demonstrated to protect cardiomyocytes and reduce mortality during the acute phase of myocardial ischemia (AMI), those strategies exhibit inadequate repair of lesion sites, as well as the process of tissue remodelling at the chronic phase, which lasts months or years and potentially increases the risk of chronically infarcted myocardium and heart failure [5, 7]. It is urgent to further dissect the biological mechanism that is involved in myocardial ischemia at the chronic phase (CMI), which might dramatically improve the prognosis of cardiovascular diseases.

As a key cofactor for various proteins and enzymes, copper plays vital roles in numerous biological processes, including energy metabolism, immune responses, angiogenesis and extracellular matrix formation [8, 9]. The impact of copper on cardiac structure and function has garnered significant attention in cardiovascular medicine [10, 11, 12, 13]. Recent studies indicate that insufficient dietary copper alters both the structure and function of the myocardium, thus leading to cardiac hypertrophy transitioning to heart failure [14]. In hypertrophic cardiomyopathy, adequate copper from the diet is proved to have promising effects in improving contractile function [15, 16]. Mechanistically, copper supplementation facilitates the activation of cytochrome *c* oxidase (C*c*O) and superoxide dismutase (SOD), which are crucial for mitochondrial metabolism in myocardium [17, 18]. As the essential element for lysyl oxidase (LOX) activity, copper also regulates collagen gridding and deposition in the myocardium to maintain extracellular matrix homeostasis [19]. Moreover, copper is required for the formation of hypoxia‐inducible factor‐1 (HIF‐1) complex to subsequently bind with the hypoxia response elements in effecting on target genes, such as angiogenic genes [20]. In clinical practice, autopsy results have revealed lower copper contents in the hearts from donors who suffered from myocardial infarction (MI) [21]. According to the fact that most of the above evidence is obtained from disease progression at the acute phase, the mechanisms underlying copper deficiency in chronic ischemic hearts remain an enigma.

Actually, the flux of copper is controlled by transporters and chaperone proteins, which are crucial for maintaining copper homeostasis with inter‐ and intra‐cellular trafficking manners [17, 22]. Indeed, abnormal expression of copper transporters, including ATP7A, ATP7B and copper metabolism MURR domain 1 (COMMD1), is confirmed to be associated with the onset of multiple diseases [8, 23, 24, 25, 26, 27, 28]. The mutation of ATP7A leads to irregular copper transportation within intestinal cells and its release into the bloodstream; thus, it induces Menkes disease [23]. ATP7B mutation could cause a rare genetic disorder named Wilson's disease, characterised by copper accumulation in various organs, particularly, the liver and brain [24]. COMMD1 is involved in hepatic biliary copper excretion by directly interacting with ATP7B [25, 26]. In knockdown murine models, the liver‐specific deficiency of COMMD1 is the leading cause of in situ copper accumulation [25, 26, 27, 28]. Nevertheless, whether the abnormal expression of those copper transporters is responsible for copper loss in the CMI progression remains unclear, especially in large primate models that are highly relevant to humans.

In our current study, rhesus monkeys were employed to construct a CMI model by permanent ligation of the left anterior descending (LAD) coronary artery without reperfusion and cardiac tissues were harvested 4 months after surgery. The results demonstrated that ischemic cardiomyocytes exhibited significantly increased copper efflux with the elevation of COMMD1, but not ATP7A or ATP7B. Further investigation demonstrated that the elevation of COMMD1 is regulated by the X‐linked inhibitor of apoptosis (XIAP), a copper‐binding protein with E3 ubiquitin ligase activity, rather than by transcriptional mechanisms.

## 2 Materials and Methods

### 2.1 Animals and Animal Care

Male Rhesus monkeys ( *Macaca mulatta* ), aged 2–3 years and weighing 4.5–6.0 kg, were obtained from Chengdu Ping‐An experimental animal breeding and research centre, a Chinese government‐accredited non‐human primate centre in Sichuan province. Eight‐week‐old male mice were obtained from DaShuo company. The animals were acclimatised to laboratory conditions for a period of at least 1 month (monkey) or 2 weeks (mouse) in the Association for Assessment and Accreditation of Laboratory Animal Care accredited facility. Animal care and experimental procedures closely followed the descriptions published previously [27, 28, 29]. All animal procedures were approved by the Institutional Animal Care and Use Committee (IACUC) at Sichuan University West China Hospital and Sichuan Cancer Hospital, following the guidelines of the US National Institutes of Health.

### 2.2 Induction of Myocardial Ischemic Infarction

All rhesus monkeys were randomly divided into the following groups: Sham‐operated control (Sham, *n* = 4), chronic phase of myocardial ischemia (CMI for 4 months, *n* = 4) and acute phase of myocardial ischemia (AMI for 2 h, *n* = 2 and AMI for 24 h, *n* = 2). As described in our previous studies [29], the rhesus monkeys of the MI group were subjected to permanent ligation of the LAD coronary artery. The sham‐operated controls were subjected to the same surgical procedure without LAD ligation. Briefly, rhesus monkeys underwent intubation after anaesthesia was induced via intravenous infusion of fentanyl, midazolam, propofol and vecuronium. Anaesthesia was maintained with continuous infusion of fentanyl and propofol via an infusion pump. Noninvasive monitoring, including electrocardiography, cuff blood pressure, pulse oximetry and capnography, was performed, while intravenous catheters were placed. The heart was exposed through a left fourth intercostal thoracotomy (4–5 cm incision). The LAD artery was occluded for 1 min followed by 5 min of reperfusion, with this occlusion‐reperfusion cycle repeated three times before eventual permanent ligation. Then the pericardium, sternum and skin were closed. For recovery, tramadol was administered intramuscularly as an analgesic. After spontaneous breathing resumed, the endotracheal tube was removed and the incision was covered with sterile gauze and bandages to ensure proper healing.

All mice were randomly divided into the following groups: Sham‐operated control (Sham, *n* = 4) and acute phase of myocardial ischemia (AMI for 6 h, *n* = 4 and AMI for 12 h, *n* = 4). As described in our previous studies [27, 28], the mice of the MI group were subjected to permanent ligation of the LAD coronary artery without reperfusion. The sham‐operated controls were subjected to the same surgical procedure without LAD ligation. Briefly, mice were anaesthetised with 4% isoflurane (R510‐22, RDW Life Science) mixed with 0.5 L/min 100% O^2^, followed by endotracheal intubation. Anaesthesia was then maintained at 2% isoflurane with 0.5 L/min 100% O^2^ during the procedure. Through a left thoracotomy between the third and fourth intercostal spaces, the heart was exposed and the LAD artery was permanently ligated using a 7/0 silk suture. The presence of ST segment elevation confirmed successful LAD occlusion.

### 2.3 Cardiac Function Analysis by Echocardiography

All monkeys were sedated with an intramuscular injection of ketamine (10 mg/kg) and midazolam (0.2 mg/kg). As previously described, transthoracic two‐dimensional echocardiography was performed on the rhesus monkeys in the left lateral position, using a 10S transducer (Vivid7 Dimension, GE Medical System) to evaluate the cardiac function at 4 months post‐MI. Standard apical two‐ and four‐chamber views were obtained, recording three consecutive cardiac cycles. Left ventricular ejection fraction (EF) was calculated using Simpson's single‐plane method, based on direct measurements of left ventricular end‐diastolic volume (EDV) and end‐systolic volume (ESV); EF = (EDV – ESV)/EDV × 100%.

### 2.4 Tissue Preparation

Rhesus monkeys were anaesthetised with ketamine, euthanised with potassium chloride by intravenous injection and sacrificed to harvest heart tissues. Mice were euthanised with isoflurane inhalation (4%) followed by cervical dislocation and heart tissues were harvested. Each heart was divided into two parts: infarct area (IA) and remote area (RA). The IA can be distinguished from the RA by its pale appearance and stiff touch. Several pieces of heart samples were preserved in liquid nitrogen for Western blotting, RT‐PCR, Co‐IP and copper concentration detection. The remaining LV tissue samples were embedded in OCT (Leica, Germany) for frozen sections and fixed with 4% paraformaldehyde for paraffin sections. The frozen sections were cut into 5 μm thick slides for immunofluorescence (IF) detection and the paraffin sections were cut into 2 μm thick slides for HE, Masson's trichrome staining and immunohistochemistry (IHC).

### 2.5 Measurements of Copper Concentrations

Heart samples stored at −80°C were lyophilized under a vacuum and weighed 10 mg of dried tissue was added to 1 mL of nitric acid (HNO_3_, Sigma, USA) and digested at 60°C overnight. Copper concentrations were determined by graphite furnace atomic absorption spectrophotometry (ICE3500, Thermo, USA) and normalised by dry tissue weight.

### 2.6 Western Blotting Analysis

The total protein levels were determined by SDS‐PAGE. Heart tissues were lysed in RIPA buffer (Beyotime Biotechnology, China) supplemented with a 1% complete EDTA‐free protease inhibitor cocktail (Roche Diagnostics, Germany). Total protein concentration was determined using the BCA protein assay kit (Thermo Pierce, USA). An equal amount of 30 μg protein was separated by 10% SDS‐PAGE and incubated in Coomassie brilliant blue solution (CBB, 0.1% R 250, 25% isopropanol, 10% acetic acid) for 45 min. Then the gels were de‐stained in a de‐stain solution (10% acetic acid, 7% ethanol) overnight. Stained gels were imaged by Vilber Fusion and analysed by Fusion‐Capt software (Vilber Lourmat, France).

The protein levels were determined by Western blotting. Equal amounts of 30 μg protein were separated by SDS‐PAGE and transferred to a PVDF membrane (Bio‐Rad, USA). PVDF membranes were incubated overnight at 4°C with the respective primary antibodies: monoclonal mouse anti‐CCS (1:100, SC‐55560, Santa cruz, USA), monoclonal mouse anti‐COMMD1 (1:100, SC‐166248, Santa cruz, USA), polyclonal rabbit anti‐COMMD1 (1:1000, A5174, Abclonal, China), monoclonal mouse anti‐XIAP (1:100, SC‐55551, Santa Cruz, USA), polyclonal rabbit anti‐XIAP (1:1000, NB‐100‐56,183, Novus, USA), monoclonal rabbit anti‐ATP7A (1:1000, ab308524, abcam, USA), monoclonal rabbit anti‐ATP7B (1:1000, ab131208, abcam, USA), monoclonal mouse anti‐GAPDH (1:1000, TA‐08, Zhongshan, China) and monoclonal rabbit anti‐p300 (1:1000, 86,377, CST, USA). Then, the membranes were incubated for 1 h at 37°C with the appropriate secondary antibody (1:1000, Santa Cruz, USA). Target proteins were visualised using a chemiluminescence HRP substrate (Millipore, USA), imaged by Vilber Fusion (Vilber Lourmat, France) and analysed by Fusion‐Capt software (Vilber Lourmat, France).

### 2.7 Reverse Transcription and Real‐Time PCR

Total RNA was extracted using Trizol (Invitrogen, USA) according to the manufacturer's instructions. Two steps were performed. First, 1 mg of total RNA was reverse‐transcribed into complementary DNA (cDNA) using a Prime Script RT reagent kit (TaKaRa, Japan). Next, cDNA was amplified by a Multicolor Real‐time PCR Detection system (Bio‐Rad, USA) with a SYBR green PCR kit (Bio‐Rad, USA) and primers for COMMD1 and XIAP. The primer sequences were designed by a Primer‐BLAST of NCBI and synthesised by Invitrogen. Gene expression levels were analysed relative to TBP.

### 2.8 IF Analysis

Frozen sections were fixed in 4% paraformaldehyde and blocked with 1% BSA at 37°C for 1 h. The slides were incubated overnight at 4°C with respective primary antibodies: monoclonal mouse anti‐CCS (1:50, SC‐55560, Santa cruz, USA), monoclonal rabbit anti‐ATP7B (1:200, ab131208, abcam, USA), polyclonal rabbit anti‐COMMD1 (1:400, A5174, Abclonal, China), polyclonal rabbit anti‐XIAP (1:100, NB‐100‐56,183, Novus, USA), monoclonal mouse anti‐Vimentin (1:500, ab8978, Abcam, USA) or monoclonal mouse anti‐α‐actinin (1:100, ab90421, Abcam, USA). Then, the slides were incubated with FITC‐labelled anti‐rabbit and TRITC‐labelled anti‐mouse secondary antibodies (Thermo, USA). The nuclei were stained by DAPI (Sigma, USA). Fluorescence images were collected by confocal microscopy (Nikon, Japan).

### 2.9 IHC Analysis

Paraffin sections were deparaffinised in easy tissue clearant and rehydrated in gradient alcohol. Then the sections were blocked by 3% hydrogen peroxide at room temperature for 10 min and 1% bovine serum albumin at 37°C for 1 h. The slides were sequentially incubated with primary antibody (monoclonal mouse anti‐CCS, 1:50, SC‐55560, Santa Cruz, USA or monoclonal rabbit anti‐ATP7B, 1:200, ab131208, abcam, USA) overnight at 4°C, HRP‐labelled secondary antibody (anti‐mouse, 1:1000, #7076, CST, USA or anti‐rabbit, 1:1000, #7074, CST, USA) at 37°C for 1 h and freshly mixed diaminobenzidine (DAB, 8059, CST, USA) according to the manufacturer's instructions. The nuclei were counterstained with haematoxylin. IHC images were randomly collected using a microscope (Eclips 80i, Nikon, Japan).

### 2.10 Co‐Immunoprecipitation (Co‐IP)

Co‐IP of COMMD1 and XIAP in monkey heart tissues was performed using a Pierce Co‐IP kit (26,149, Thermo, USA) according to the manufacturer's instructions. The monoclonal mouse anti‐COMMD1 (SC‐166248, Santa Cruz, USA) or mouse IgG_3_ was incubated with AminoLink Plus Coupling Resin or Pierce Control Agarose Resin on a rotator at room temperature for 120 min. Heart tissues were lysed in ice‐cold IP Lysis/Wash Buffer. The total protein of the lysates was measured by the BCA protein assay kit (Thermo Pierce, USA). A total of 1 mg of lysates (250 μL, 4 mg/mL) was incubated with the antibody‐coupled resin on a rotator overnight at 4°C. The resin was washed with elution buffer and the protein complexes bound to the antibody were eluted. To make a 1× final solution, 5× sample buffer was added to the elution buffer and heated at 100°C for 10 min. Then Western blotting analyses were performed as described before. XIAP was detected by a polyclonal rabbit anti‐XIAP (1:1000, NB‐100‐56183, Novus, USA) and COMMD1 was detected by a polyclonal rabbit anti‐COMMD1 (1:1000, A5174, Abclonal, China).

### 2.11 Statistical Analysis

GraphPad Prism (GraphPad Software, USA) was applied to statistical analysis. All data were presented as mean ± SEM. One‐way ANOVA was used for the statistical evaluation of sham and different areas of the MI group, followed by the Dunnett *t* test. Student's *t* test was used to compute statistical significance between two groups. The values of *p < 0.05, **p < 0.01 and ***p < 0.001 were considered statistically significant.

## 3 Results

### 3.1 Copper Reduction in Cardiomyocytes Facilitates CMI Progression of Rhesus Monkey

**FIGURE 1:**
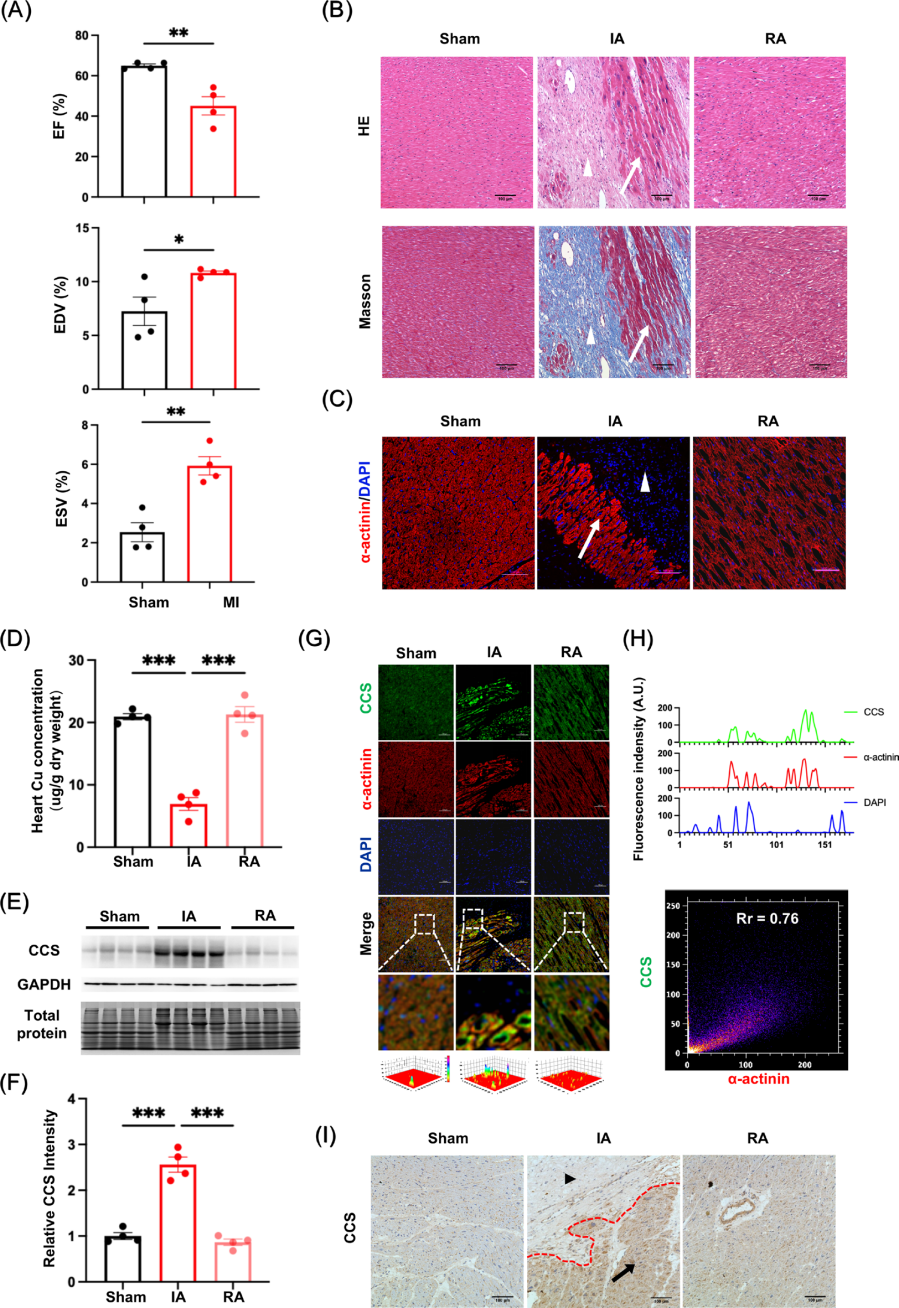
The copper concentration was decreased in the ischemic cardiomyocyte of rhesus monkeys. (A) The myocardial contractile functions of ejection fraction (EF%), end‐systolic volume (ESV) and end‐diastolic volume (EDV) measured by echocardiography. (B) Images of HE, Masson's trichrome staining and (C) immunofluorescence of α‐actinin (cardiomyocytes marker) in the heart section of rhesus monkeys, the white arrowhead showed the survival cardiomyocytes and the white triangle showed the collagen (Masson staining) and fibroblasts (HE and IF staining). (D) The copper concentration of hearts was analysed by AAS. (E) Western blot and (F) quantitative analysis of the protein level of CCS in different portions of hearts. (G) Immunofluorescence image, 3D‐heat map and (H) co‐localization analysis of CCS (green) and α‐actinin (red, cardiomyocytes marker) in the hearts, DAPI stain (blue) labels nuclei. (I) Immunohistochemistry analysis of CCS in the hearts, the black arrowhead indicates the survival cardiomyocytes zone and the black triangle indicates the fibrotic zone. Scale bar = 100 μm, data (Sham: *N* = 4; MI: *n* = 4) were expressed as mean ± SEM. *p < 0.05, **p < 0.01, ***p < 0.001 versus Sham control. IA, infarct area; RA, remote area.

As one of the most well‐recognised animal models for cardiopathy, the heart of primates exhibits comparable functional features and anatomic structures when compared with human [30, 31, 32, 33, 34, 35]. Therefore, we utilised rhesus monkeys to construct the CMI model by permanent ligation of the LAD coronary artery. Four months post‐surgery, cardiac contractile functions were assessed for CMI in monkeys to evaluate the long‐term effects of ischaemia on cardiac function. As shown, a 33% reduction in EF was observed in the surgery group. Meanwhile, a 57% increase in EDV with a 1.4‐fold increase in ESV was monitored in CMI animals compared to sham‐operated controls (Figures 1A and S1A). These results indicated a constitutive impairment in cardiac functions at CMI. Pathologically, other than extensive cardiomyocyte death in the ischaemic myocardium, inflammatory infiltration and substantial collagen deposition were found in the same regions of CMI monkeys (Figure 1B). Following IF analysis identified a decrease in α‐actinin, which is considered the marker of cardiomyocytes (Figure 1C). This reduction of cardiomyocytes was further confirmed by the sustained apoptosis as indicated by the enhancement of cleaved‐Caspase‐3 levels (Figure S1B,C).

Due to the critical roles of copper homeostasis in MI [8, 9, 10, 11, 12, 13, 14], we speculated that copper might affect the loss of cardiomyocytes in our CMI model. Thus, copper concentrations were measured in the IA and RA of the myocardium. Compared to sham‐operated controls and RA, a significant downregulation of copper levels was exclusively observed in the IA (Figure 1D). Meanwhile, as a candidate copper indicator, copper chaperone for superoxide dismutase (CCS) responded inversely to changes in copper status and was found to increase in the IA (Figure 1E,F). By employing both IF and IHC staining, we confirmed that the elevated CCS co‐localised with cardiomyocytes in the lesion site of the CMI monkey (Figure 1G–I). Taken together, on the basis of the successfully established CMI monkey model, we revealed copper deficiency in ischemic cardiomyocytes, which is responsible for cell loss, thus leading to constitutive cardiac dysfunction.

### 3.2 Elevated COMMD1 Is Associated With Copper Efflux in Ischemic Cardiomyocytes During CMI Progression

**FIGURE 2:**
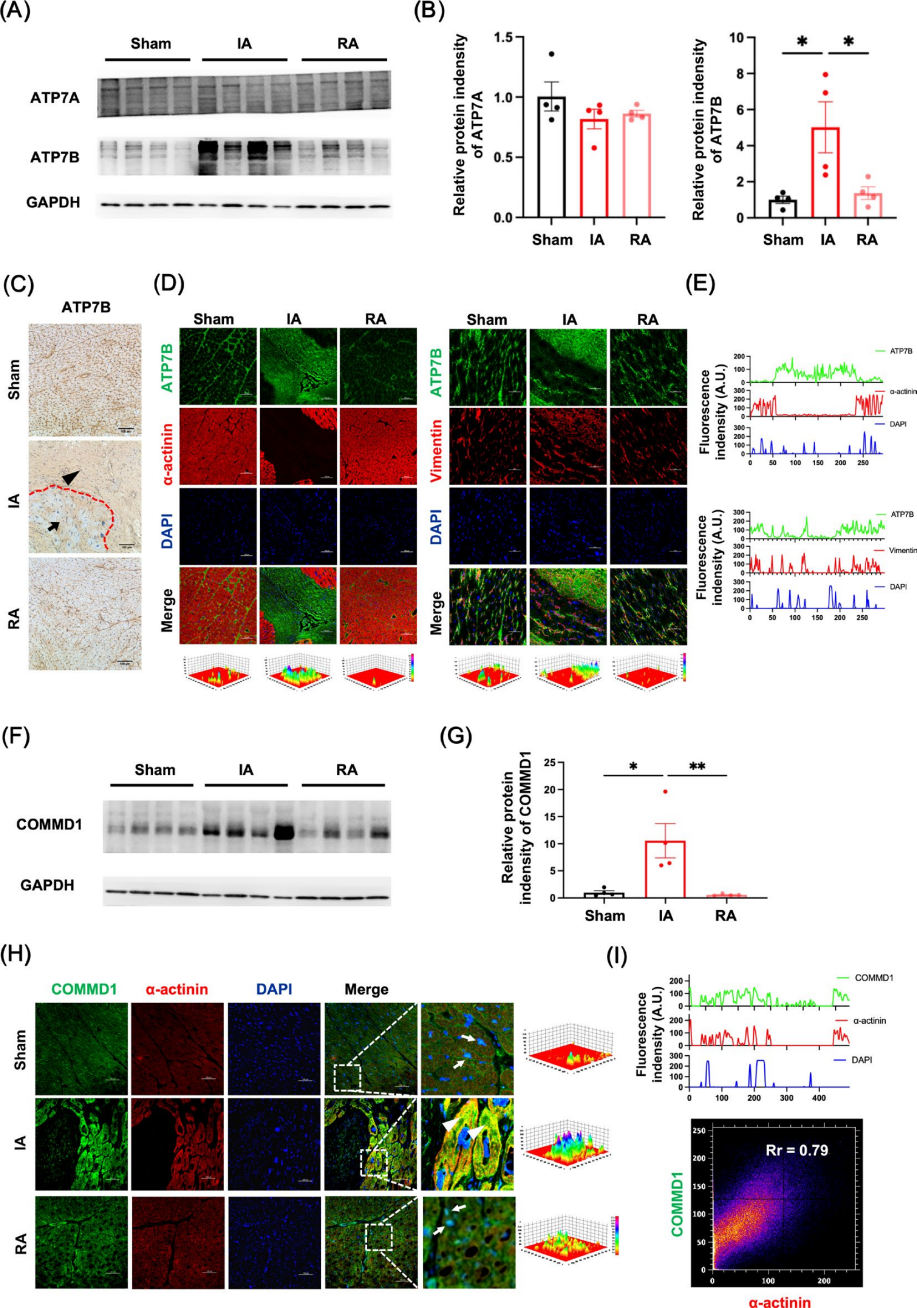
The changes of copper transporters in the chronic ischemic myocardium of rhesus monkeys. (A) Western blot and (B) quantitative analysis of the protein levels of ATP7A and ATP7B in different portions of hearts. (C) Immunohistochemistry analysis of ATP7B in the hearts, the black arrowhead indicates cardiomyocytes and the black triangle indicates fibroblasts. (D) Immunofluorescence image, 3D‐heat map and (E) co‐localization analysis of ATP7B (green) with α‐actinin (red, cardiomyocytes marker) or vimentin (red, fibroblast marker) in the hearts, DAPI stain (blue) labels nuclei. (F) Western blot and (G) quantitative analysis of the protein levels of COMMD1 in different portions of hearts. (H) Immunofluorescence image, 3D‐heat map and (I) co‐localization analysis of COMMD1 (green) with α‐actinin (red, cardiomyocytes marker) in the hearts, DAPI stain (blue) labels nuclei, the white arrowhead indicates nuclear localised COMMD1 and the white triangle indicates cytoplasmic localised COMMD1. Scale bar = 100 μm, data (Sham: *n* = 4; MI: *n* = 4) were expressed as mean ± SEM. *p < 0.05, **p < 0.01 versus Sham control. IA, infarct area; RA, remote area.

Copper transporters play critical roles in regulating ionic uptake and efflux, essential for maintaining copper homeostasis in cardiomyocytes. To investigate the mechanism of copper depletion in ischemic cardiomyocytes, we analysed the expression patterns of ATP7A and ATP7B, both of which promote copper efflux during chronic ischemia. As shown in Figure 2A,B, the protein level of ATP7A remained consistent in both RA and IA. In contrast, ATP7B showed a remarkable elevation in IA compared to RA and sham‐operated controls (Figure 2A,B). Meanwhile, IHC was employed to additionally demonstrate the association between ATP7B expression and MI (Figure 2C). Besides, IF results indicated that increased ATP7B protein mainly co‐localised with vimentin+ fibroblasts in the lesion site, but not α‐actinin^+^ cardiomyocytes from IA and RA of the CMI monkey model, or sham‐operated controls (Figure 2D,E). Given the decisive role of COMMD1 in orchestrating P‐type ATPases transportation from cytoplasm vesicles to membrane, as well as the copper efflux [36, 37], the change of COMMD1 was examined in the ischemic myocardium of rhesus monkeys. Accordingly, upregulation of COMMD1 was found in the IA compared with the RA and sham‐operated controls (Figure 2F,G). Further IF results illustrated that α‐actinin^+^ ischemic cardiomyocytes in IA were responsible for the enhanced COMMD1 protein, which also shifted to the cytoplasm from the nuclei (Figure 2H,I). Due to the effectiveness of cytoplasm COMMD1 in controlling copper efflux, our observations not only provided evidence of the correlation between COMMD1 and ATP7B in the heart, but also revealed a direct regulatory role of COMMD1 in copper efflux.

### 3.3 XIAP Is Linked to COMMD1 Elevation in Ischemic Cardiomyocytes

**FIGURE 3:**
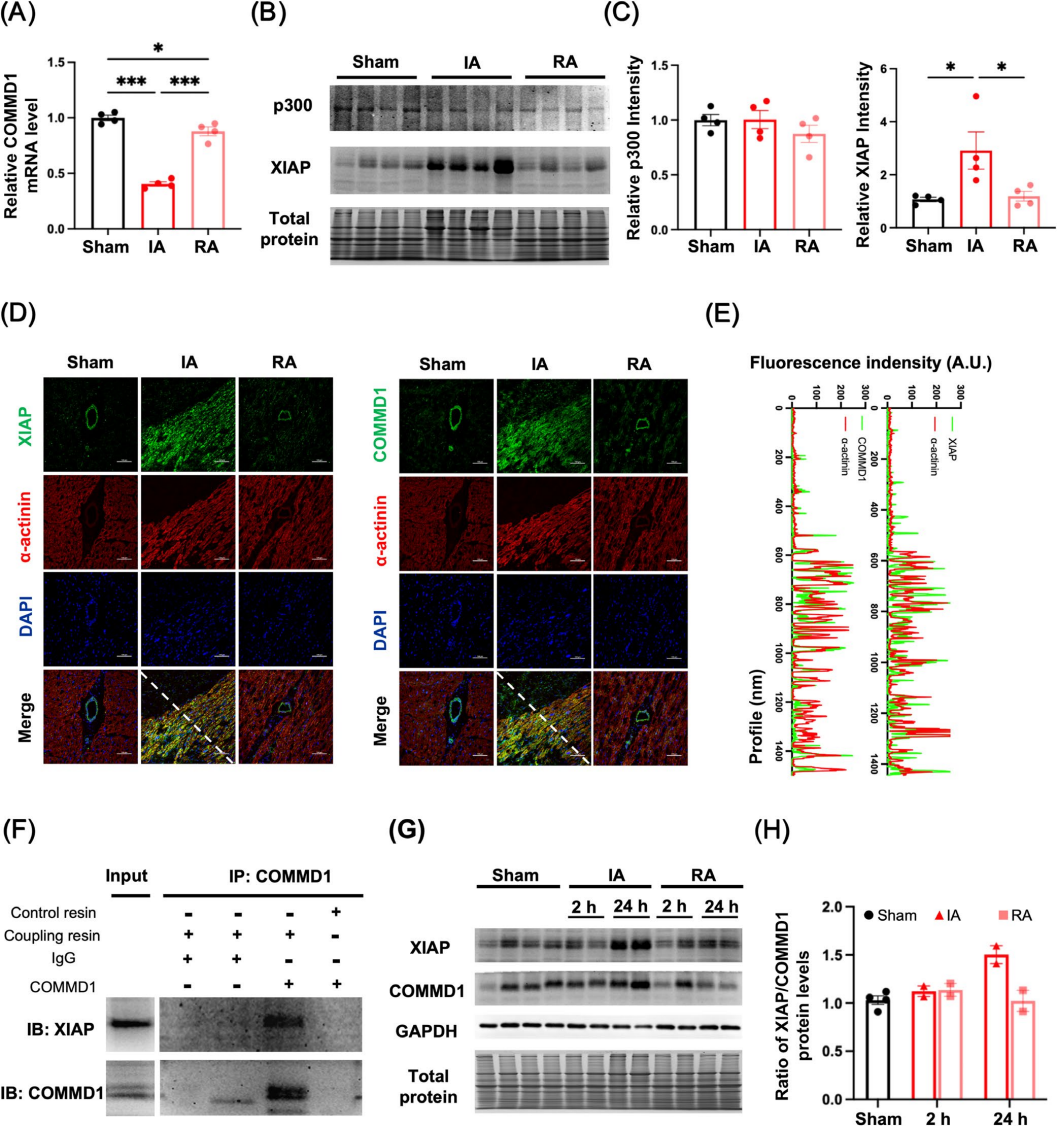
Increased XIAP associated with COMMD1 in cardiomyocytes after chronic LAD ligation in rhesus monkeys. (A) The mRNA levels of COMMD1 determined by RT‐PCR. (B) Western blot and (C) quantitative analysis of the protein levels of p300 and XIAP in different portions of the heart of rhesus monkey (Sham: *n* = 4; MI: *n* = 4). (D) Immunofluorescence image and (E) co‐localization analysis showing COMMD1 (green) or XIAP (green) with cardiomyocytes (α‐actinin, red) in the serial sections of the heart of rhesus monkey, DAPI stain (blue) labels nuclei, scale bar = 100 μm. (F) Immunoprecipitation‐Western blotting analysis of COMMD1 and XIAP in the ischemic heart tissue of rhesus monkeys and the control resin + mouse antibody against COMMD1 or coupling resin + mouse IgG as the control groups. (G) Western blotting analysis of the protein levels of COMMD1 and XIAP in the cardiac tissues of rhesus monkeys after 2 and 24 h of LAD ligation (Sham: *n* = 4; 2 h MI: *n* = 2; 24 h MI: *n* = 2). (H) The ratio of XIAP versus COMMD1 protein levels in the heart of rhesus monkeys. Data were expressed as mean ± SEM. *p < 0.05, **p < 0.01, ***p < 0.001 versus Sham control. IA, infarct area; RA, remote area.

In contrast to the protein levels, the expression of COMMD1 mRNA was downregulated in the IA compared to the RA and sham‐operated controls (Figures 2F,G and 3A). This discrepancy between mRNA and protein levels is likely due to post‐translational modifications, which regulate the stability and localisation of the COMMD1 protein. We measured the protein levels of both XIAP and p300, which are considered to mediate ubiquitination or acetylation in regulating COMMD1, respectively [38, 39]. Only XIAP was found to be significantly upregulated in the IA of rhesus monkeys with ischaemic myocardium (Figure 3B,C). Further analysis confirmed that both XIAP and COMMD1 were enriched in α‐actinin+ ischaemic cardiomyocytes of rhesus monkeys (Figure 3D,E). To determine the direct effectiveness of XIAP in modifying COMMD1 ubiquitination, Co‐IP analysis was employed and XIAP was identified as being involved in COMMD1 immunoprecipitates, but not in control IgG or control resin (Figure 3F).

Besides, temporal specificities of XIAP and COMMD1 expression were also monitored during MI progression in rhesus monkeys with post‐LAD ligation (Figure 3G,H). As shown, compared with both sham‐operated controls and RA, LAD ligation led to the enhanced protein level of XIAP in IA at 24 h, which preceded the changes in COMMD1 (Figure 3G). Meanwhile, the ratio of XIAP versus COMMD1 proteins was consistently increased in IA of rhesus monkeys with LAD ligation (Figure 3H). Considering the relatively short observation period in rhesus monkeys, we also explored the temporal relationship between elevated COMMD1 and XIAP proteins in a murine model of MI as well, and similar temporal specificities of XIAP and COMMD1 expression were examined at 6 and 12 h after surgery (Figure S2). As shown in Figure S2, elevated XIAP was observed at 6 h post‐LAD ligation, preceding the subsequent increase in COMMD1 at 12 h. Taken together, we believe the elevation of COMMD1 protein levels was largely due to the post‐translational modification by XIAP.

### 3.4 Copper Deficiency Prompted the Enhancement of COMMD1 via Post‐Translational Modification by XIAP in Ischemic Cardiomyocytes

**FIGURE 4:**
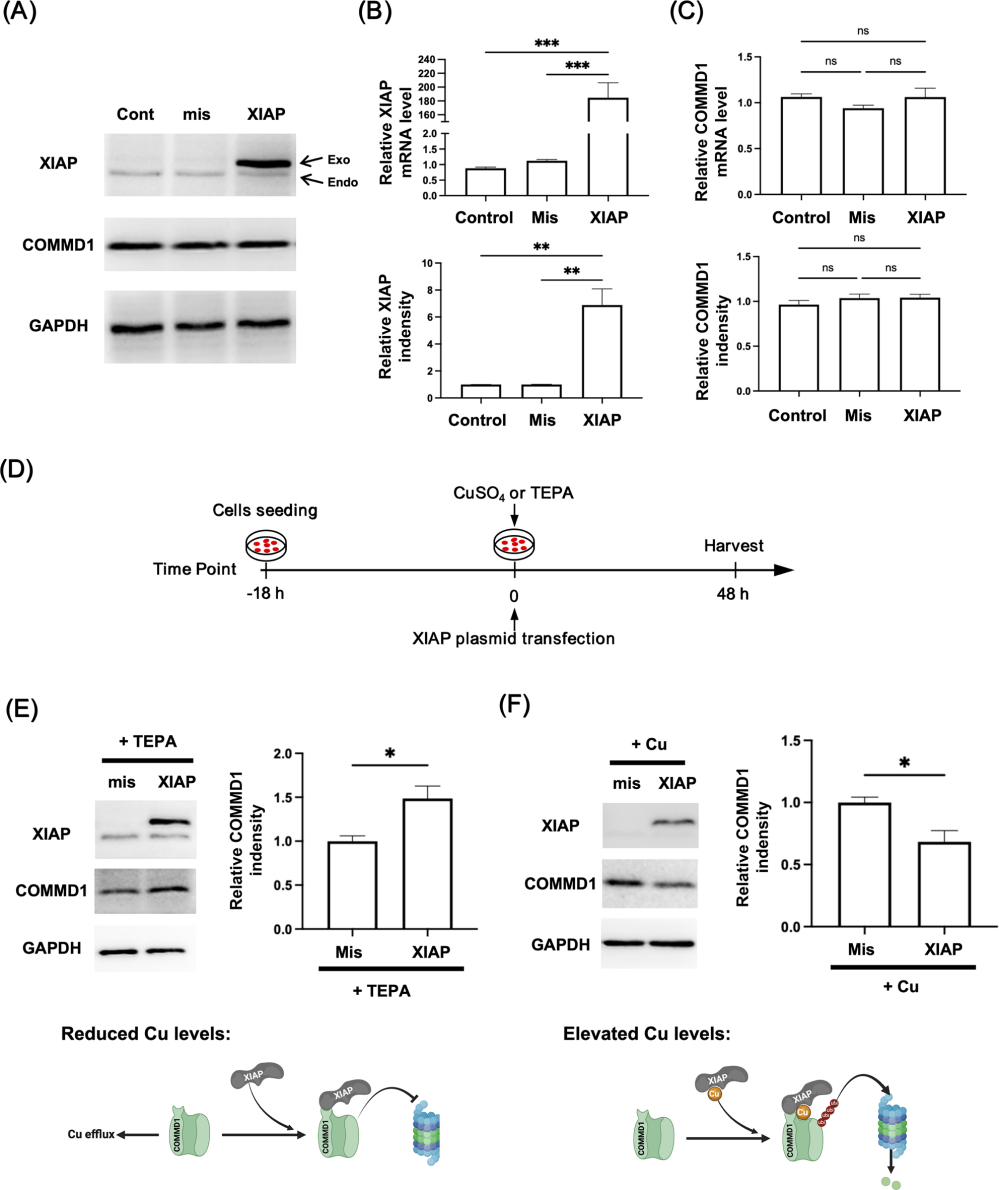
Effect of XIAP overexpression on COMMD1 levels in H9C2 cells under varying copper concentrations. (A) Western blotting analysis of the protein levels of COMMD1 and XIAP after 48 h of XIAP‐overexpression‐plasmid transfection. (B) Quantitative analysis of the mRNA and protein levels of XIAP and (C) COMMD1 after 48 h of XIAP‐overexpression‐plasmid transfection. (D) Outline for the treatment of H9C2 cells. (E) Western blot and quantitative analysis of the protein levels of COMMD1 after 48 h of transfection with XIAP‐overexpression‐plasmid and treatment with 50 μM TEPA. (F) Western blot and quantitative analysis of the protein levels of COMMD1 after 48 h of transfection with XIAP‐overexpression‐plasmid and treatment with 50 μM CuSO_4_. Data (*n* = 3) were expressed as mean ± SEM. *p < 0.05, **p < 0.01, ***p < 0.001 versus mismatch group.

To uncover the role of post‐translational modification by XIAP, the rat cardiomyocyte cell line H9C2 was employed to construct XIAP constitutive expression. Accordingly, both mRNA and protein levels of XIAP were detected to elevate due to ectopic expression, but COMMD1 was undisturbed at both transcription and translation levels, indicating that XIAP improvement alone was insufficient to trigger post‐translational modification (Figure 4A–C). Considering that XIAP is also a metalloprotein with a strong affinity for copper, it might initiate the ubiquitin‐dependent pathway [36]. TEPA (copper chelator) or CuSO_4_ was used to treat H9C2 cells with exogenous XIAP expression (Figure 4D). Based on the TEPA‐mediated copper deletion in H9C2 cells at 48 h, exogenous XIAP significantly induced the expression of COMMD1 protein (Figure 4E). By contrast, excessive copper from 50 μM CuSO_4_ not only reduced COMMD1 protein levels but also affected both endogenous and exogenous expression of XIAP in H9C2 cells (Figure 4F). Other than the expected regulatory mode of COMMD1, we speculated that a cell apoptotic procedure was promoted to diminish XIAP expression as a disruption of copper homeostasis [40, 41, 42]. All above, by binding with XIAP, copper might act as a key metal ion in bringing alternative post‐translational modulation of COMMD1.

## 4 Discussion

**SCHEME 1:**
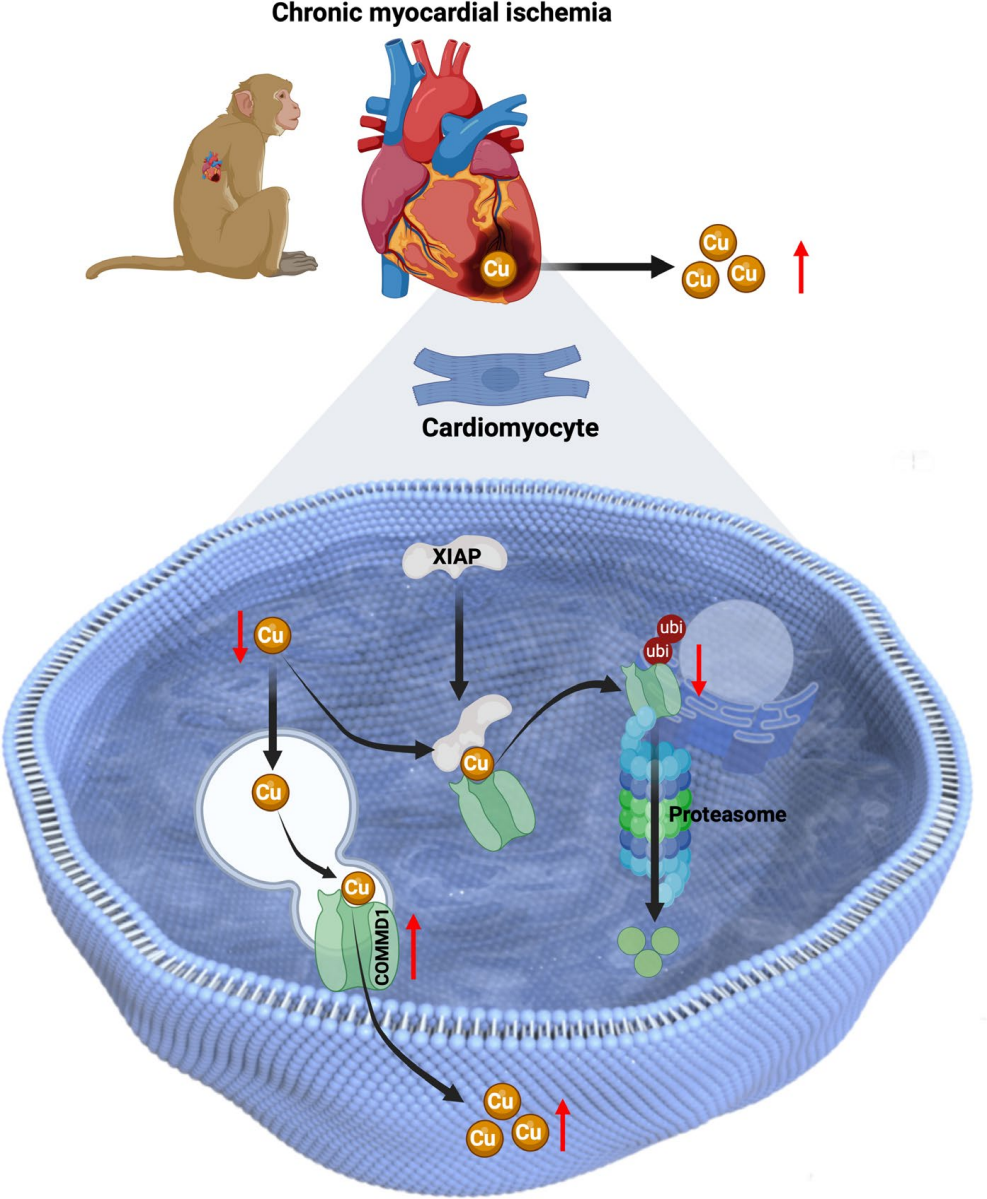
Elevated COMMD1 contributes to cardiomyocyte‐copper efflux during chronic ischemic hearts of rhesus monkeys, regulated by XIAP through post‐translational modification.

By establishing ischemic myocardium in rhesus monkeys, both functional and pathological changes were evaluated to demonstrate that the primate model could preferably mimic the cardiac symptoms of human patients with CMI. In revealing a copper efflux‐related apoptosis of ischemic cardiomyocytes (Figure 1), elevation of COMMD1 levels was detected in cardiomyocytes that are responsible for copper efflux, but not P‐type ATPases transporters (ATP7A and ATP7B) (Figure 2). Rather than transcriptional regulation, this enhancement of COMMD1 protein was confirmed to be primarily mediated by post‐translational modification via XIAP signalling (Figure 3). Meanwhile, copper efflux was also involved in promoting XIAP‐initiated post‐translational modification of COMMD1 protein, thus accelerating the progression of CMI (Figure 4). Therefore, our findings not only propose a potentially positive feedback loop among XIAP, COMMD1 and copper efflux to orchestrate ischemic cardiomyocyte loss, but also provide novel therapeutic strategies for preventing the development of CMI (Scheme 1).

In current mechanism studies of MI, rodents are the most well‐recognised animal models due to their convenient manipulation at both the cellular and genetic level [14, 15, 16, 27, 28]. However, the discrepancies between primates and rodents in heart rates and cardiac ultrastructure largely restrict the following translational researches [43, 44]. Accordantly, non‐human primate models, particularly, rhesus monkeys, provide us with an opportunity to mirror clinical symptoms and pathological changes of human patients with MI owing to their resemblance patterns of physiological signals, cardiac anatomy and coronary vasculature [33, 34, 35]. Meanwhile, unlike the compensatory recovery in rodent models, both human patients and rhesus monkeys are confirmed to exhibit progressive inflammation, metabolic disorder and fibrosis to obstruct ventricular remodelling after MI [33, 34, 35]. These characteristics allow us to perform longitudinal studies in investigating the long‐term development of CMI, especially the decisive role of XIAP‐COMMD1 signalling for cardiomyocyte apoptosis [29]. In addition, by modifying copper concentration to disrupt ion homeostasis, we generated an easy‐access way to inhibit XIAP‐COMMD1 signalling, thus protecting rhesus monkey cardiomyocytes, which could be considered a pre‐clinical trial for therapeutic strategies of CMI.

CCS is widely used as an indicator of intracellular copper status due to the phenomenon of copper‐induced CCS degradation [45, 46, 47, 48]. Under conditions of high intracellular copper concentration, CCS could bind and transfer copper ions to copper‐dependent enzymes, such as SOD1 and XIAP. At the same time, CCS undergoes ubiquitination at lysine 48 (K48) by XIAP, targeting it for proteasomal degradation [46, 48]. In contrast, the interaction of copper‐free CCS and XIAP results in nondegradative ubiquitination of CCS at lysine 241 (K241), which prevents CCS degradation when intracellular copper levels are low [47, 48]. Given that changes in CCS expression are useful for monitoring copper status in cells, we investigated the expression changes and distribution of CCS in the heart of CMI monkeys, as well as copper concentration measurements by atomic absorption spectrophotometry. Our findings revealed an elevation of CCS at protein levels in ischemic cardiomyocytes, which could possibly indicate a significant depletion of intracellular copper in ischemic cardiomyocytes of monkeys with CMI. Overall, CCS upregulation might reflect copper loss at the lesion site of CMI monkeys, thus highlighting the disrupted copper homeostasis in ischemic cardiomyocytes.

As an essential element, copper plays vital roles in various biological processes, including C*c*O in mitochondrial electron transport, SOD in antioxidant defence, HIF‐1 in angiogenesis and LOX in collagen cross‐linking [8, 9, 13, 49]. Previous studies revealed that these processes are critical for the regulation of cardiac metabolism and contractile function [15, 16, 17, 18, 19]. Nevertheless, cardiac copper efflux has been documented in both human and animal studies; copper supplementation is shown to protect cardiac contractile function in MI of the rhesus monkey [50]; however, the mechanism of copper efflux in CMI remains unclear. Using a primate model, our study dissected that copper deficiency localised in ischemic cardiomyocytes, accompanied by extensive apoptosis, collagen deposition and impaired cardiac function in CMI of rhesus monkeys. Given these important roles of copper in maintaining cardiac function, our findings further indicated a potential therapeutic method for targeting copper deficiency during CMI. On the other hand, COMMD1 is reported to directly interact with ATP7B in facilitating hepatic copper handling, whereas by interacting with ATP7A, COMMD1 is involved in the process of copper transport during the gastrointestinal tract and across the blood–brain barrier [25, 26]. Therefore, ATP7A and ATP7B play distinct roles in copper transport by binding with COMMD1 [23, 24, 25]. Although copper homeostasis is tightly regulated by copper transporters, ATP7A showed no ischemia‐responsive changes in the heart [17, 22]. Meanwhile, unregulated ATP7B was found in fibroblasts and increased COMMD1 was specifically localised in cardiomyocytes, suggesting a unique COMMD1‐mediated copper efflux mechanism in ischemic cardiomyocytes that is independent of ATP7A or ATP7B.

In this study, given the distinct expression patterns between mRNA and protein levels, we identified that post‐translational modification is the principal mechanism underlying the elevated COMMD1 levels in ischemic hearts of rhesus monkeys. Previous studies indicated that XIAP facilitates COMMD1 ubiquitination at lysine 48 (K48) to promote proteasomal degradation [38], while p300‐mediated acetylation protects COMMD1 from XIAP‐induced degradation to stabilise its post‐translational levels [39]. And these alternative post‐translational regulatory roles of XIAP were mainly determined by copper homeostasis [38, 48, 51, 52]. Consistently, our current studies revealed an XIAP elevation in cardiomyocytes with COMMD1 expression from the rhesus monkey of ischemic myocardium. This temporal pattern of expression suggested that the rise in COMMD1 protein followed XIAP elevation, implicating XIAP as a key regulator of COMMD1 under ischemic conditions. However, under copper‐deprivation conditions, excessive XIAP was monitored to enhance COMMD1 protein levels in cardiomyocytes, whereas saturated copper reduced COMMD1 under XIAP overexpression. We speculated that XIAP might serve as a metalloprotein undergoing substantial conformational change with high affinity for copper‐binding, thus potentially activating the ubiquitin‐dependent pathway [38, 48, 51, 52], which is further supported by the inefficient post‐translational modification of COMMD1 by XIAP under copper homeostasis. Our findings suggest that copper acts as a critical ion to trigger alternative post‐translational modulations of COMMD1 by XIAP. In summary, we propose a positive feedback loop wherein copper deficiency promotes XIAP elevation, which in turn stabilises and increases COMMD1 expression through post‐translational modification, potentially exacerbating copper efflux in ischemic cardiomyocytes. This phenomenon highlights the intricate interplay between XIAP and COMMD1 in regulating cardiac copper efflux, as well as sheds light on further investigation in therapeutic strategies to manage copper homeostasis in CMI.

In summary, our study demonstrated the cause‐and‐effect relationship between copper loss from the ischemic myocardium and copper transporters. As the copper transporter, COMMD1 is primarily identified to function in copper excretion of liver [24, 25, 26]. With COMMD1 deficiency, Bedlington terriers and murine models were observed to suffer hepatic copper toxicosis [24, 25, 26]. However, the role of COMMD1 in regulating copper efflux under pathological conditions remains unclear. By establishing chronic MI in rhesus monkeys, we revealed that COMMD1 contributes to the loss of copper in cardiomyocytes during CMI. Considering the decisive interaction of ATP7A or ATP7B with COMMD1 to regulate copper transport [23, 24, 25, 37], we investigated both localization and expression levels of ATP7A and ATP7B in our current setting. Circumstantially, sustained expression of ATP7A was monitored during disease progression, whereas ATP7B localised in other cardiac cell types than cardiomyocytes, which showed a COMMD1 upregulation in cardiomyocytes. These findings prompted us to hypothesise that COMMD1‐mediated copper efflux in ischemic cardiomyocytes may occur independently of ATP7A and ATP7B. In addition, the loss of copper ions dampened XIAP‐induced COMMD1 ubiquitination and degradation in ischemic cardiomyocytes and overexpression of XIAP alone failed to prevent copper efflux in cardiomyocytes. This novel positive feedback loop not only highlights the essential role of copper ions in CMI development but also illustrates a potential reason for progressive aggravation in human patients.

### 4.1 Limitations of the Study

Due to the obstacle of genetic modifications in primates, as well as the lack of proper targeted inhibitor and/or agonist, it is extremely challenging for us to further investigate the therapeutic strategies by targeting COMMD1 or XIAP in rhesus monkeys with CMI. As reported, XIAP negatively regulates COMMD1 by forming K48 polyubiquitin chains in promoting ubiquitination and proteasomal degradation in 293 cells [38, 53]. The current study observed an interaction between XIAP and COMMD1 and the following exploration unveiled the role of XIAP in regulating the posttranslational modification of COMMD1 with copper iondependence. Unfortunately, the nature of the presumed posttranslational modification of COMMD1 in hearts is not fully defined in our study. These are indeed the limitations of our study and further exploration of this aspect will be needed in subsequent research.

## Author Contributions

C.L., C.X. and N.D. performed the experiments. X.L., X.F. and J.Y. helped design experiments. D.L. and C.L. analysed the data. C.L., X.Y. and C.X. wrote the manuscript. M.Y. revised the manuscript. All authors have read and agreed to the published version of the manuscript.

## Conflicts of Interest

The authors declare no conflicts of interest.

## Supporting information


**Data S1.** Supporting Information.

## Data Availability

All data supporting the study findings can, upon reasonable request, be made available from the corresponding author.
