# Supplementary material for: Elevated COMMD1 Contributes to Cardiomyocyte Copper Efflux in Chronic Myocardial Ischemia: Insights From Rhesus Monkey
Source: Cell Prolif. 2025 Mar 3;58(8):e70016. doi: 10.1111/cpr.70016 (PMC12336456; doi:10.1111/cpr.70016)
Supplement: Supplementary file 1 — Data S1. Supporting Information. [file CPR-58-e70016-s001.docx]

**Supplementary information**

**Elevated COMMD1 Contributes to Cardiomyocyte Copper Efflux in Chronic Myocardial Ischemia: Insights from Rhesus Monkey**

Chen Li^1†^, Da Li^2†^, Xia Cheng^1†^, Xiaoli Yuan^2^, Ning Du^3^, Xin Liao^1^, Xiaorong Feng^1^, Jie Yao^1^, Chenglong Li^4*^, Chengxia Xie^5*^, Mu Yang^1*^

^1^ Department of Experimental Research, Sichuan Clinical Research Center for Cancer, Sichuan Cancer Hospital & Institute, Sichuan Cancer Center, University of Electronic Science and Technology of China, Chengdu, Sichuan, China

^2^ Department of General Internal Medicine, Sichuan Clinical Research Center for Cancer, Sichuan Cancer Hospital & Institute, Sichuan Cancer Center, University of Electronic Science and Technology of China, Chengdu, China

^3^ Chengdu Customs Technology Center, Chengdu, China

^4^ Department of Pharmacy, Deyang People’s Hospital, Affiliated Hospital of Chengdu University of Traditional Chinese Medicine, Deyang, China

^5^ Department of Laboratory Medicine, West China Hospital, Sichuan University, Chengdu, China

^†^ The authors contribute equally

* Correspondence: chenglongsmile@126.com, chengxia_xie@163.com, mu.yang@uestc.edu.cn


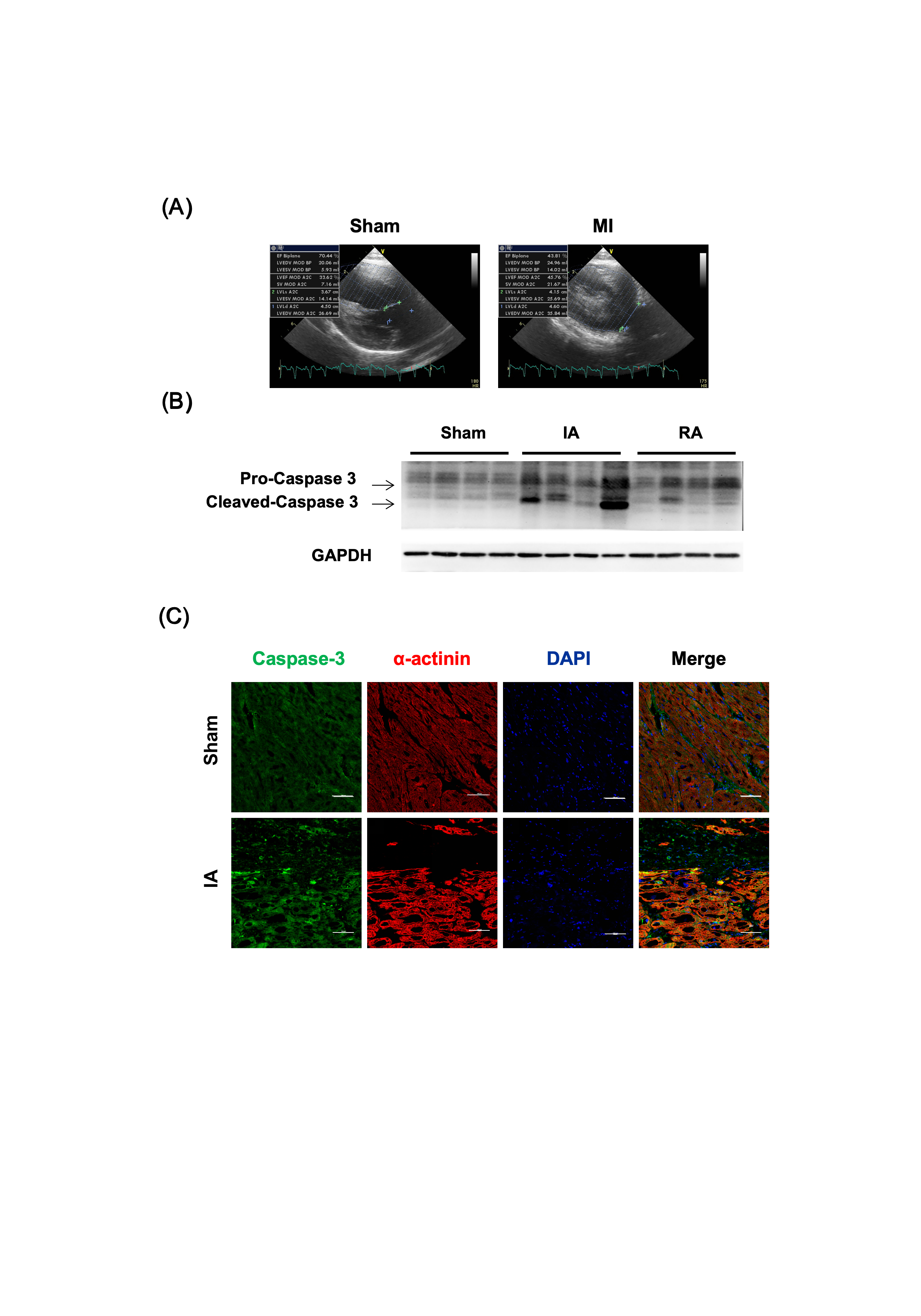


**FIGURE S1** (A) Echocardiographic detection of changes in cardiac function. (B) Western blot analysis of the protein level of Caspase-3 in different portions of hearts. (C) Immunofluorescence image and co-localization analysis of Caspase-3 (green) and α-actinin (red, cardiomyocytes maker) in the hearts, DAPI stain (blue) labels nuclei.

**
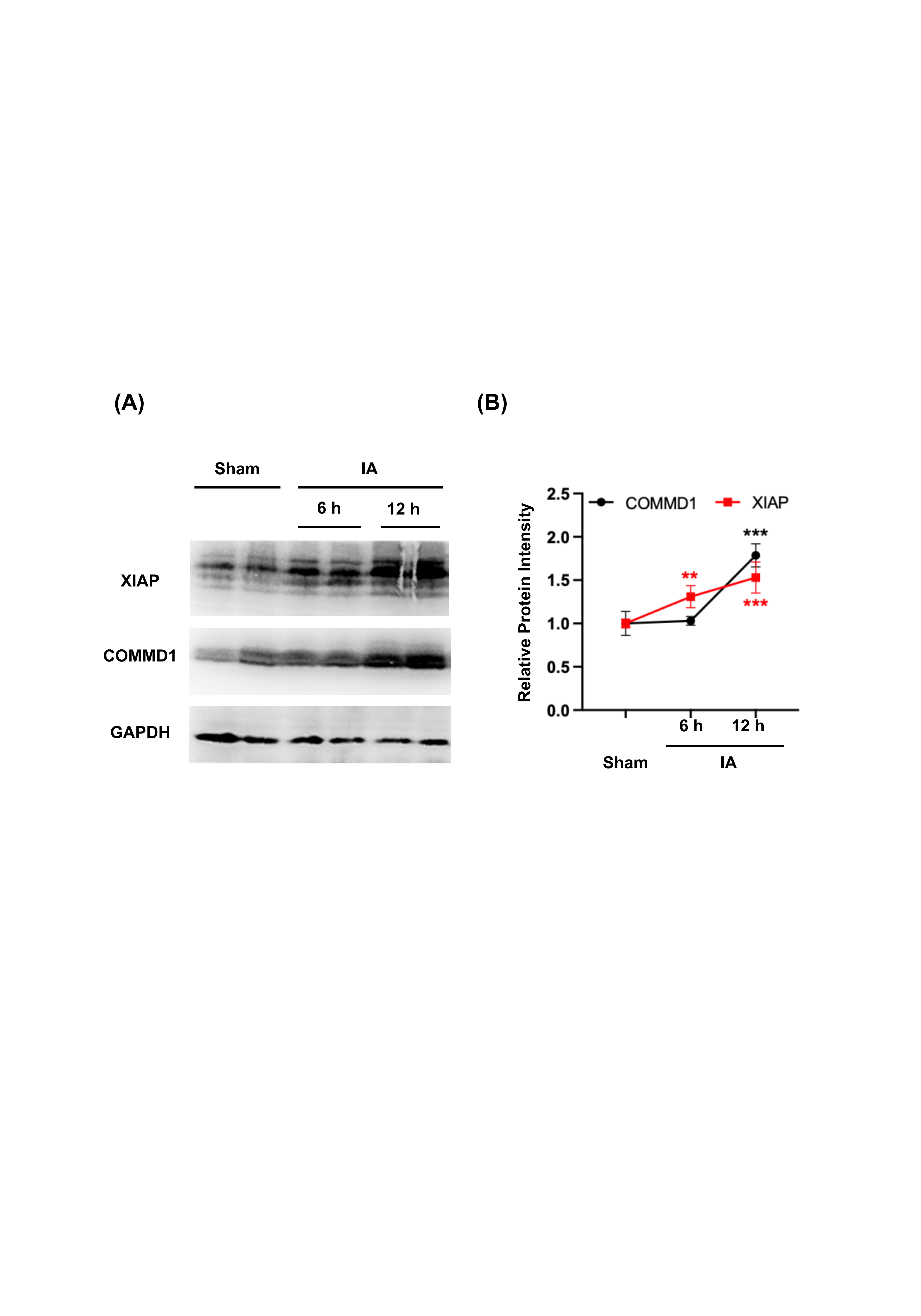
**

**FIGURE S2 The upregulation of XIAP preceded the increase of COMMD1 in the ischemic myocardium of mice.** (A) Western blotting and (B) quantitative analysis of the protein levels of COMMD1 and XIAP in the cardiac tissues of mice after 6 and 12 hours of LAD ligation (Sham: n=4; 6h IA: n=4; 12h IA: n=4). Data were expressed as mean ± SEM, ***p* < 0.01, ****p* < 0.001 versus Sham control, IA: infarct area.
